# Supplementary material for: Ex vivo lung CT findings may predict the outcome of the early phase after lung transplantation
Source: PLoS One. 2020 May 29;15(5):e0233804. doi: 10.1371/journal.pone.0233804 (PMC7259648; doi:10.1371/journal.pone.0233804)
Supplement: Attachment — Submitted filename: Response to Reviewers.docx [file pone.0233804.s001.docx]

**Academic editor: Your MS has been highlighted by expert reviewers as novel and interesting, and therefore I invite you to thoroughly revise your paper according to the very helpful comments of my colleagues. Looking forward to seeing your revised MS.**

Answer:

We would like to thank the editors for the review and the opportunity to revise our manuscript. We have carefully revised our paper according to all comments of the reviewers. Details of our revisions are outlined further below in a point-by-point response to the reviewers.

**Reviewer #1: This is an interesting study, examining pre-implant CT scans of donor lungs. Previous similar reports have been of low numbers, so this is a novel study.**

**The references to EVLP are irrelevant, and could be removed – this is a completely separate examination, and does not need to be compared with EVLP.**

Answer:

We agree with reviewer #1’s comments. We have removed all sentences related to EVLP from the manuscript.

**It is made clear only in the discussion that** **the transplant was already underway when the CT was performed, so the results did not alter the decision to proceed. This information should come earlier in the manuscript.**

Answer:

This is a very good point. We have revised the “Patients and Methods.” It is now clear that the transplant was already underway when the CT was performed and we did not alter the decision to proceed.

**We are given results only in the cases undergoing CT scan. The data should be put in the context of the overall transplant programme. How did the donor choices and recipient outcomes differ, if at all, in the lungs not subjected to CT scan? How was the decision to perform the scan made? Were there different donor features?**

Answer:

We thank reviewer #1 for pointing that out. Actually, EVL-CT has been done in all lung transplant cases except living-donor lung transplant cases since October 2007 in our hospital. We have added the information in “Patients and study groups.”

**The donor demographics do not contain donor blood gases, a widely used marker of donor quality. Is this data available? Did it differ in lungs with infiltrates?**

Answer:

This is a very good point. We have added the data of donor arterial blood gasses to Table 1 and Table 2, and revised the “Results” and “Discussion.”

**The data comes from over 10 years of activity. How was it distributed in this time span? Ie were a lot of the scans done in the later part of the series? This again highlights the need to put the scanned patient in the whole context of the series.**

Answer:

We thank reviewer #1 for the positive feedback. As we mentioned above, EVL-CT has been done in all lung transplant cases except living-donor lung transplant cases. At least in our hospital, EVL-CT is already an important option in our clinical practice.

**There is a simple division of those lungs with infiltrates and those without. Whilst numbers were small, was any other analysis made of the infiltrates? Were they quantified? Was there any pattern linked to any particularly less good outcome?**

Answer:

We appreciate reviewer #1’s comments. We tried quantitative analysis of the infiltrates using software; however, it was impossible. In terms of the simple division of the lungs, we have added some comments in “Patients and study groups” on how we analyzed the nature of infiltrates.

**The microbiology results are intriguing and potentially important. How were** **the bronchial aspirates obtained? Do they amount to a BAL, which properly samples** **organisms in the lung parenchyma? If so, was this guided by the CT? Or was it just an upper airway aspiration, where the findings are, in other series, less important. We presume this was not just a suction down the endotracheal tube in the donor, so effectively a tracheal rather than bronchial aspirates?**

Answer:

We agree with reviewer #1’s comments. Unfortunately, the bronchial aspirates obtained in this study do not amount to a BAL. We have toned down the comments on bronchial aspirates in “Discussion.”

**Reviewer #2: This is a retrospective clinical study by investigators from Tohoku University in Sendai, Japan, investigating the value of ex-situ CT imaging of donor lungs immediately prior to lung transplantation.**

**The authors have investigated the correlation between abnormal findings on chest CT and early outcome after transplantation. They concluded that ex-situ CT imaging may help in evaluating donor lungs prior to acceptance for transplantation.**

**Major comments:**

**1) Study period:**

**The authors studied 70 donor lung pairs over a period of 11 years (2007-2017). It would be interesting to know how many lung transplants were performed at their centre during the study period.**

**- Did all donor lungs have CT imaging prior to transplantation? (70 transplants in eleven years is a very low annual number)**

**- If not, how were the donor lungs selected to undergo CT imaging?**

**- What happened to donor lungs that were already rejected for transplantation at the donor hospital? Where these lungs also recovered for CT imaging?**

**- please discuss the selection of donor lungs in the paper and provide a study flow chart describing all donor offers over the study period with the number of lungs recovered, lungs scanned ex-situ with CT, and lungs transplanted.**

Answer:

We appreciate reviewer #2’s comments. The total number of lung transplants in the period is actually a bit small, 92 transplants. We have revised the “Patients and study groups” and mentioned how we selected the patients in this study. We excluded living-donor lung transplantation (10 cases), lobar lung transplant (6 cases) and retransplantation (6 cases) from this study. The number of 92 transplants is still low if compared to other transplant centers in North America and European countries. However, in Japan, this number is not really bad and our hospital is one of the largest transplant centers.

We did not recover lungs that were rejected at the donor hospital. As we mentioned earlier, we have revised the “Patients and Methods” according to reviewer #1’s comments. It was not clear the study flow. We hope that it is now clear in the manuscript that the transplant was already underway when the CT was performed and we did not alter the decision to proceed and that the results of EVL-CT did not alter our clinical practice.

**2) Terminology:**

**The authors have used the term “extracted donor lung (EDL) CT”. The word “extracted” is not well choosen. The reviewer would prefer the term “ex-situ” donor lung CT imaging in contrast to “in-situ” donor lung imaging while the lungs are still inside the deceased donor body.**

**- please change the terminology throughout the paper.**

Answer:

We completely agree with this comment. The word “extracted” is obviously inappropriate term for describing the technique. We have chosen “ex vivo” instead of “extracted.”

**3) CT scoring and reading by radiologist:**

**It is well known that reading of CT images is subjective with an important inter-observer variability.**

**- How were the CT readers trained to describe findings of donor lungs on images taken outside the human body?**

**- How many radiologist were involved in the study? How many looked at the images of 1 pair of donor lungs? Was there consensus on the findings amongst radiologists?**

**- How did they do the scoring? Have they used a scoring system? How were the donor lungs assigned to one of both study groups?**

**- Were the radiologist blinded to the macroscopic findings by the surgeon who retrieved the lungs?**

**- Were there any lungs that were declined after reading the CT images?**

**- Beside “infiltration”, some of the donor lungs will have some underlying parenchymal disease (emphysema, interstitial fibrosis); Nothing is mentioned about parenchymal lung disease in the paper. See also paper by Gauthier et al (reference below). Was any underlying lung disease observed during imaging? Were these lungs excluded for transplantation?**

**Please describe in the methods in more details the technique and the number of radiologists involved in this study. Please discuss findings of underlying lung disease.**

Answer:

We appreciate reviewer #2’s comments on the reading of CT images. All CT readers were trained in advance of the study and we revised “Patients and study groups.”

As the reviewer #2 commented the way to divide the recipients between the control and infiltration group is one of the most important parts in this study. We have significantly revised the part regarding the CT reading in “Patients and study groups.”

In terms of the declines, in our current practice, we do not decide the availability of the lung graft based on the EVL-CT data. In other words, when CT images are obtained, the lung was already accepted for transplantation and the transplant surgery was already underway. As we mentioned above, we received similar comments from reviewer #1.

**4) PGD grading:**

**The ISHLT PGD grading system was first published by Christie et al in 2005 and then modified by Snell et al in 2017. The paper by Barr M et al (reference 4) does not discuss the PGD grading system!**

**- Please describe which PGD grading system (2005 vs 2017) was used to score the lungs after transplantation and refer to the correct paper.**

Answer:

We thank the reviewer for noting this flawed reference. It was an error. We used the PG grading system by Christie et al in 2005.

**5) Sensitivity and specificity of donor X-ray and CT scan:**

**The authors discuss in the paper that some of the donors had in-situ imaging in the days before retrieval. It is well known that standard X-ray imaging suffers from a low sensitivity and low specificity for abnormal findings. E.g. for the BLT group, the false positive rate was 13% and the false negative was 54.5% (see table 1). The accuracy of CT scan should be better. However, the false positive rate of CT scan in this series was still 66.7% (4/6; see Table 1).**

**- the authors should discuss** **in this paper the sensitivity and specificity of standard X-ray and CT imaging based on other papers in the literature and correlate this with their own findings in the study.**

**- it is important to stress that the cause of a “radiologic infiltrate” in donor lungs ranges from “pneumonia” to simple “atelectasis”. Abnormal findings on the CT scan should always be checked inside the donor prior to declining a donor offer!! (see paper by Martens et al Eur J Cardiothorac Surg. 2016 Nov;50(5):832-833; Accepting donor lungs for transplant: let Lisa and Bob finish the job!)**

Answer:

We appreciate reviewer #2’s favorable comments. We added the paper by Vanstapel et al. (“Histopathologic and radiologic assessment of nontransplanted donor lungs”) to the references. We think that the paper supports the importance of CT imaging of a donor. We also referred to the paper by Vanstapel et al. and discussed the sensitivity and specificity of standard X-ray in this paper.

We referred to the paper by Verleden et al. (“Radiological Analysis of Unused Donor Lungs: A Tool to Improve Donor Acceptance for Transplantation?” Am J Transplant. 2017:1912-1921.) and revised the “Discussion” as well as “Introduction.” We discussed the infiltrates in the EVL-CT.

**6) References:**

**The authors briefly discuss 4 papers (out of a total of only 10 references) whereby ex-situ MRI was used to correlate findings. These papers do not discuss the use of CT and do no refer to lung transplantation. The reviewer believes that all 4 references should be deleted.**

**On the other hand, there are more papers discussing the use of CT scan to evaluate donor lungs in-situ and ex-situ!**

**- please discuss the following papers in the revised version:**

**Real-Time Computed Tomography Highlights Pulmonary Parenchymal Evolution During Ex Vivo Lung Reconditioning. Sage E, De Wolf J, Puyo P, Bonnette P, Glorion M, Salley N, Roux A, Liu N, Chapelier A. Ann Thorac Surg. 2017 Jun;103(6):e535-e537. doi: 10.1016/j.athoracsur.2016.12.02**

**Chest computed tomography imaging improves potential lung donor assessment. Gauthier JM, Bierhals AJ, Liu J, Balsara KR, Frederiksen C, Gremminger E, Hachem RR, Witt CA, Trulock EP, Byers DE, Yusen RD, Aguilar PR, Marklin G, Nava RG, Kozower BD, Pasque MK, Meyers BF, Patterson GA, Kreisel D, Puri V. J Thorac Cardiovasc Surg. 2019 Apr;157(4):1711-1718.e1. doi: 10.1016/j.jtcvs.2018.11.038.**

**Commentary: A plea for a donor CT! Hoetzenecker K. J Thorac Cardiovasc Surg. 2019 Apr;157(4):1720-1721. doi: 10.1016/j.jtcvs.2018.12.039. Epub 2018 Dec 21.**

Answer:

We really appreciate reviewer #2’s comments. We agree with the suggestion that the 4 papers whereby ex-situ MRI was used to correlate findings should be deleted. We have revised the “Discussion” substantially and added the 3 papers that the reviewer recommended. Now the number of references has increased.

**Minor comments:**

**7) Figure legends:**

**- Figure 2 and Figure 3: please correct the word “transactional” into “transaxial”**

**- Figure 6 B: ICU stay was not significantly different in SLT! Please correct the word “significantly” in the sentence!**

**- Figure 7: please refer in the legend to the correct reference for PGD grading.**

Answer:

We thank the reviewer for noting these flawed words and sentences. We have now revised all these points. We have added the reference for PDG grading.

**8) Tables 7& 8:**

**Both tables can be deleted; the data can be summarized in the text in the results section.**

Answer:

We completely agree with the comment. We have deleted Table 7 and 8.

**9) Figure 7:**

**The current figure is difficult to interpret the differences between both groups. The reviewer believes that a survival curve showing “freedom from PGD3” would better** **depict the differences over time (T0-T72)** **between the control and the infiltration group.**

Answer:

We thank reviewer #1 for the suggestion and have revised Figure 7 accordingly. Now the figure depicts well the difference in the recovery from PGD between the control and the infiltration group.

**Reviewer #3: The objective of this study was to investigate the correlation between EDL-CT findings and post-transplant outcome in patients who underwent bilateral lung transplantation (BLT) or single lung transplantation (SLT).**

**Below are some comments:**

**1. The nature of the study was exploratory. Allocation of control or infiltration group was not random and might depend on some variables which might or might not be directly recorded. Was there any methods applied to take into account such heterogeneity? Methods to compare the 2 groups with adjustments should be considered.**

Answer:

We think that what reviewer #3 pointed out is quite right. We tried to compare the 2 groups with adjustments, for example with propensity score matching. However, it was impossible due to the small number of patients. We have added some comments on this in the “Discussion” as a limitation.

**2. Lines 234-235: As acknowledge by the authors due to the non-intervention nature, this study could not** **demonstrate the real practical usefulness of EDL-CT. How would you use the current information on EDL-CT from this study? Is there any plan for future research?**

Answer:

We completely agree with reviewer #2’s comment. We could not demonstrate the real practical usefulness of EDL-CT because of the study design. In the current practice, as we presented a case in Figure 4, we can just obtain findings that cannot be detected by preretrieval chest x-ray. However, we believe that such information is useful under some circumstances. For instance, even when we would like to check chest CT scans of a recipient after lung transplantation, it is often difficult to transfer the recipient to the medical imaging center if the patient is on ECMO. Currently, we obtain the information of EVL-CT of the lung graft in every donor and it often helps us to understand the findings of posttransplant chest x-rays.

In terms of future plans, we have added some future plans in “Discussion.”

**3. In the article, it’s not clear what was the primary endpoint for the study. Given the small sample sizes in both cohorts, the test statistics and the observed p-values should be interpreted carefully (including statements on significant difference etc). Apart from PGD and ICU mortality, has other endpoints such as a 30 mortality rate had ever been considered?**

Answer:

We agree with this comment. The sample size of this study is small and we have to be careful in interpreting the differences. We think that this is one of the limitations of the study. In terms of the 30-day mortality, in the series of the recipients in the study, there was no 30-day mortality case.

**4. How was the sample size and the power for this study determined? This should be clarified. If it is a convenient sample size, reasons to support such case should be underlined.**

Answer:

We simply included all the cases with EVL-CT data except living-donor lung transplantations (10 cases), lobar lung transplantations (6 cases) and retransplantations (6 cases). We have revised “Patients and study groups.”

**5. The PGD grade at each time point was analyzed by repeated measures two-way ANOVA. As in point 1, please explain how the heterogeneity can be handled with this technique. Do you consider an alternative model to take into account heterogeneity of the different baseline factors when conducting this analysis?**

Answer:

We appreciate reviewer #3’s comments. As we mentioned above, the heterogeneity is one of the weak points in this study. We have stated the limitation in “Discussion.” Instead of repeated measures two-way ANOVA, we tried to use Kaplan-Meier curves and statistically compare using the Mantel-Cox log-rank test. But, again, we do not think that we can overcome the problem of the heterogeneity.

**6. Lines: 226-227: ‘CT. We believe that EDL-CT may predict the post-transplant outcome in lung transplantation.’ Please provide evidence for this statement. Do you have any supporting data in the current study?**

Answer:

We have toned down the conclusions made in the first version of the paper. We think that EVL-CT may predict very early after lung transplantation: only post-transplant ventilation and ICU stay periods.
