# Supplementary material for: Ex vivo lung CT findings may predict the outcome of the early phase after lung transplantation
Source: PLoS One. 2020 May 29;15(5):e0233804. doi: 10.1371/journal.pone.0233804 (PMC7259648; doi:10.1371/journal.pone.0233804)
Supplement: Attachment — Submitted filename: Response to Reviewers-ver.3.docx [file pone.0233804.s002.docx]

**Academic editor: I do agree with reviewer 3 to include a statement about the lack of sample size calculation, and to add a CONSORT diagram (patient seleciton/study groups). This will make the MS much more clear.**

Answer:

We would like to thank the editors for the review and the opportunity to revise our manuscript. We have added a statement that we did not perform sample calculation. In addition, we made a consort diagram (Fig 2).
